# Investigation on Flexural Behavior of Geopolymer-Based Carbon Textile/Basalt Fiber Hybrid Composite

**DOI:** 10.3390/polym13050751

**Published:** 2021-02-28

**Authors:** Chi Hiep Le, Petr Louda, Katarzyna Ewa Buczkowska, Iva Dufkova

**Affiliations:** 1 Department of Material Science, Faculty of Mechanical Engineering, Technical University of Liberec, Studenstká 2, 461 17 Liberec, Czech Republic; petr.louda@tul.cz (P.L.); katarzyna.ewa.buczkowska@tul.cz (K.E.B.); iva.dufkova@tul.cz (I.D.); 2 Department of Materials Technology and Production Systems, Faculty of Mechanical Engineering, Lodz University of Technology, Stefanowskiego 1/15, 90-924 Lodz, Poland

**Keywords:** textile-reinforced geomortar, chopped basalt fibers, carbon textile, Charpy impact strength, flexural strength, failure mode

## Abstract

This paper presents an experimental research on the mechanical properties of the hybrid composite thin-plates of the short basalt fibers (CBFs)/carbon textile-reinforced geomortar. The effect of fiber contents and lengths of CBFs on the flexural behavior of carbon textile-reinforced geopolymer specimens (TRGs) was investigated by the four-point flexural strength and Charpy impact test. The experimental results of hybrid TRGs, on the one hand, were compared with reference TRGs, without CBF addition; on the other hand, they were compared with the results of our previous publication. According to the mixing manner applied, fresh geomortar indicated a marked reduction in workability, increasing the CBF loading. Furthermore, using CBFs with lengths of 12 mm and 24 mm makes it easy to form the fiber clusters in geomortar during mixing. According to all the CBF loadings used, it was found that TRGs showed a significant improvement in both static and dynamic flexural strength. However, the failure mode of these TRGs is similar to that of the reference TRGs, described by the process of fiber debonding or simultaneously fiber debonding and collapse. In comparison with our prior work results, neither the CBF dose levels nor the fiber lengths used in this work have yielded a positive effect on the failure manner of TRGs. According to the results of the Charpy impact test, this reveals that the anchoring capacity of textile layers in geomortar plays an important role in specimens’ strength.

## 1. Introduction

Textile-reinforced concrete (TRC) is a composite construction material resulting from a combination of a fine-grained cementitious matrix and textile materials. Textile reinforcements could be made of synthetic fibers, such as alkali-resistant glass, basalt, carbon, polymer, natural fibers, etc. [1,2,3,4]; this makes it possible to produce the thin-ply composites which do not require minimum concrete covering layer as corrosion protection, as compared to steel reinforcement. Due to its excellent mechanical properties, TRC can be applied in the production of thin-walled façade elements, load-bearing integrated framework, tunnel lining, or in the strengthening of existing/new reinforced concrete structures [5,6,7,8].

The mechanical properties of TRC depend on two key factors, including the mechanical properties of its components and the bonding strength between the fiber yarns and cementitious matrix. The matrix-fiber interfacial bond directly affects the mechanical performance of TRC, since it resists crack opening and crack propagation. Many studies have been investigated to improve the bonding strength of fiber yarns to matrix, such as impregnation epoxy [9,10], coatings with quartz sand [9,11], impregnation with nanoparticles [12], adding short fibers [10,11,13,14,15,16,17,18,19], surface treatment [20,21], and pre-tension [16,17,19].

Adding short fibers can be considered one of the most convenient methods to improve the mechanical performance for both cementitious matrix and the matrix-fiber interface, since they are added directly to the mixture. Deju Zhu et. al. [15] reported the tensile behavior of TRC with five basalt textile layers and various contents of short carbon, steel, and glass fibers. They found that first-crack stress, ultimate load, and toughness are significantly improved by short fibers; however, the enhancing trend differs by increasing the concentrations of various short fibers. In addition, short glass and steel fibers have caused an increase in the number of cracks by reducing the crack spacing and width, while short carbon fibers have no obvious effect. Rabea Barhum et. al. [18] carried out uniaxial tensile tests of alkali-resistant glass TRC containing short dispersed and short integral glass fibers; and the results indicated that the addition of short dispersed fibers has enhanced first-crack stress significantly, whereas a pronounced improvement in tensile strength was noted for the TRC composite with the addition of short integral fibers. Yunxing Du et. al. [13,17,19] addressed a series of studies on the effectiveness of short steel fibers on an uniaxial tensile, flexural loading of basalt- or carbon-fiber-based TRC. The results showed that the matrix-textile interfacial bonding improved with the addition of steel fibers; thus, the first-crack and ultimate stresses of TRC specimens were improved. By increasing the concentration of steel fibers, the number of cracks increased in TRC specimens, but the average crack spacing decreased. Yining Ding et. al. investigated the effect of polypropylene fibers on the load-bearing capacity of basalt fiber TRC. Their results confirmed that the addition of macro polypropylene fibers increased the stress in the post-peak region and changed the brittle failure mode into a ductile mode.

As outlined above, it was observed that a variety of short fibers, such as glass fiber, carbon fiber, steel fiber, polypropylene, etc., has been used as additional reinforcement to improve the mechanical performance of TRC. However, there is a lack of research on TRC with addition of short basalt fibers; and most of the studies use Portland cement as the mortar matrix for the production of TRC. This motivates us to try to make the composite thin-plates of carbon textile-reinforced geopolymer with addition of short basalt fibers and evaluate their mechanical performance.

The geopolymer concept was introduced by Joseph Davidovits in the late 1970s [22]. Geopolymers are amorphous inorganic polymer materials made of aluminosilicate materials (fly ash, metakaolin, slag, etc.) and alkali solutions [23,24,25,26]. Depending on the source materials and reasonable mixing ratio, geopolymers can exhibit a similar or enhanced performance in terms of mechanical properties and durability as compared to a Portland cement-based binder (OPC) [27,28,29,30]. They are also considered an environmentally friendly building material due to lower a CO_2_ emission in the production of the raw materials as compared to OPC. Therefore, geopolymers are considered as the new generation of green cement alternative to OPC. Fiber-reinforced geopolymers are the combination of a geopolymer binder with fibers. Heretofore, many works have been performed on geopolymer-based composites reinforced with various types of fibers, such as short fibers, unidirectional fibers, fabrics, or textiles [31,32,33,34,35,36,37,38]. On the other hand, for textile-reinforced geopolymers, most investigations have focused on their use as additional outer layers for the strengthening of structural elements [31], [39,40,41,42]; very few studies have investigated the mechanical performance of the textile/geopolymer composite itself.

In the our previous works [43,44], short basalt fibers (CBFs) at high dose levels were added to geomortar for the production of the carbon textile-reinforced geopolymer specimens (TRGs). Therefore, the flexural behavior of these TRGs was assessed only by the concentrations of CBFs. This paper describes the experimental investigation of the influence of both dosages and the fiber lengths of three types of CBFs (6 mm, 12 mm, 24 mm) on the static flexural and impact flexural resistance of TRGs. The geomortar matrix was added by different CBF concentrations of 0.25%, 0.50% and 0.75%, except for 6 mm CBFs up to 1.0%; and TRGs were produced by a combination of the carbon textile layer with the geomortars. The four-point and Charpy impact bending test were measured to assess the mechanical properties. In addition, the results obtained in this work were also compared with the results achieved in a previous work.

## 2. Materials and Methods

### 2.1. Raw Materials

**Figure 1:**
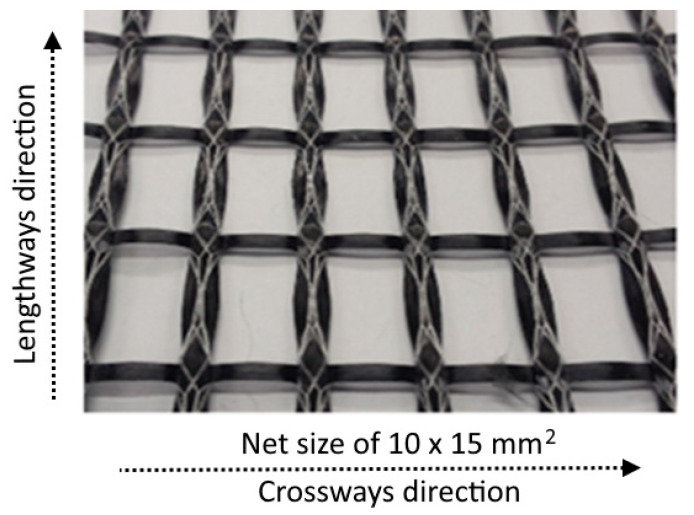
Image of textile reinforcement: carbon fiber textile with the net size of 10 × 15 mm^2^.

**Figure 2:**
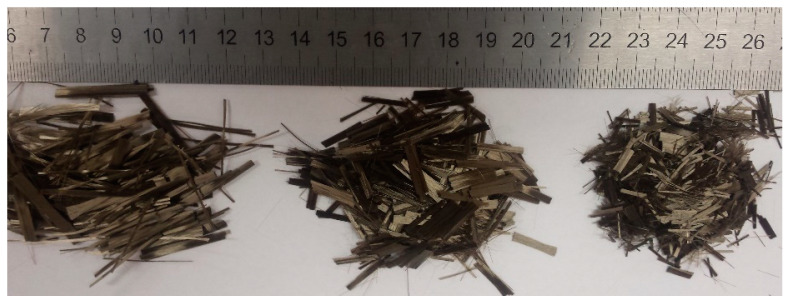
Three types of the chopped basalt fibers (CBFs) with different fiber lengths used in this work, from left to right: the fiber length of 24 mm (24 mm CBFs), the fiber length of 12 mm (12 mm CBFs), the fiber length of 6 mm (6 mm CBFs).

Metakaolin-based geopolymer cement, purchased by České Lupkové Závody, a.s., Czech Republic, acts as aluminosilicate material source for the production of the geopolymer paste (in weight percent: SiO_2_—47.4; Al_2_O_3_—29.7; CaO—14.5; MgO—2.6; TiO_2_—1.8; Fe_2_O_3_—0.5; K_2_O—0.3; Na_2_O—1). The geopaste was activated by geocement with a sodium silicate-based alkali solution of modulus 1.73 (in weight percent: SiO_2_—20.72; Na_2_O—12.33; H_2_O—66.68). Two types of quartz sands, supplied by Sklopísek Střelec, a.s., Czech Republic, act as the fine aggregates for the production of the geomortar matrix; one, with a maximum grain size of 0.063 mm, is named as micro-milled sand, whereas the second, with a grain size of 0.6–1.25 mm, is named as rough sand. Silica fume with the main component of amorphous SiO_2_ was purchased from Kema Mikrosilika–Sanační centrum, s.r.o., Sviadnov, Czech Republic. The chemical composition of the silica fume was as follows (wt. %): SiO_2_—90, CaO—0.8, MgO—max. 1.5, Al_2_O_3_—max. 1, Na_2_O—0.5. A carbon textile was used as textile reinforcement for the production of TRG, as shown in Figure 1, whereas Table 1 shows its material characteristics. The chopped basalt fibers (CBFs), purchased by Kamenny Vek, were used as additional reinforcement for the TRG composites. Three types of CBFs with different fiber lengths (~6 mm, 12 mm, and 24 mm) were applied. The CBFs have the same individual fiber diameter of 13 µm, and density of 2.67 g/cm^3^, a tensile strength of 2700–3200 MPa, and a tensile modulus of 85–95 GPa, shown in Figure 2.

### 2.2. The Mixing Process of the Fresh Geomortar and Specimens Preparation

Table 2 shows mix proportions of the geomortar matrix. Firstly, geocement and activator solution with a given ratio were mechanically stirred for about 4 min to achieve a homogenous geopaste. The geopaste is preferably mixed separately to ensure the best possible dissolution of alumina and silica in the alkali environment, which helps to promote the geopolymerization process of the geopaste as well as possible. Secondly, fine-particles reinforcement, including silica fume and micro-milled sand, were added to the slurry, and the mixture was stirred for about 3 min more. Next, CBFs were added after step 2 was completed, and the mixture was mixed for several minutes so that the fibers were evenly distributed in the geomortar, followed by the rough sand being added and mixed. After adding sand, the mixture must be mixed at a slow speed to minimize breaking the initial length of CBFs. This approach to mixing is to assess the effectiveness of the fiber length of CBFs on the mechanical properties of TRGs. The freshly prepared geomortar was cast into the molds with dimensions of 30 × 30 × 150 mm^3^. The samples were covered by polypropylene plastic film and cured at lab temperature for 28 days for the strength test. The flexural strength and compressive strength of the geomortar are shown in Table 3.

**Figure 3:**
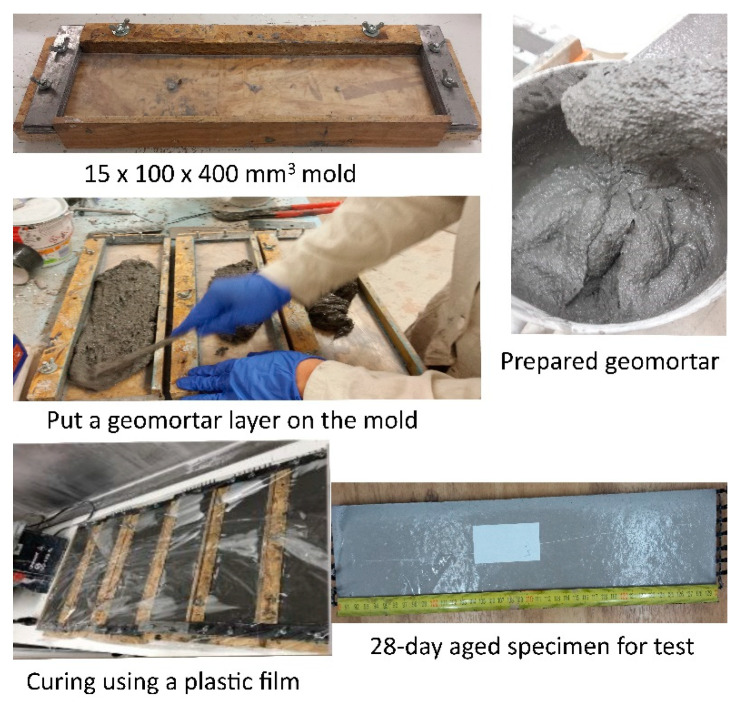
Process of preparation of the carbon textile-reinforced geopolymer (TRG) specimens for the four-point bending test.

The fresh mixture prepared above together with one layer of carbon textile was used to fabricate the TRG composites, whereas no CBFs were added to the reference TRGs. The specimens were molded in the rectangular form with the approximate dimensions of 400 × 100 × 15 mm^3^. Firstly, a geomortar layer with the desired thickness (5 mm) was poured on the bottom of the mold, followed by the placement of a textile layer. Next, a geomortar layer on the mold top was fixed to ensure the desired thickness of the tested samples. The textile layer was positioned in the mold with the desired distance by using the thin metal plates at the ends of the mold. Next, the nut bolts were tightened together to fix the thin metal plates. The mold was manually vibrated for a while to ensure the good penetration of the fresh mortar between the textile layers. Next, each fiber yarn was stretched as well as possible using the adjustable wrench in order to ensure the tension state of fiber yarns embedded in the mortar. Finally, the mold was manually vibrated again for several minutes, adding the geomortar into the mold in case it was missing. The specimens were then covered by polypropylene plastic film for 24h at the laboratory. After that, the specimens were demolded, followed by re-wrapping using the wrapping material. The specimens were further cured at the laboratory with a relative humidity of 55% and temperature of 22 °C until the test time (28 days). Figure 3 shows the process of preparation of the TRG specimens. It is noted that the number of fiber yarns per one textile layer arranged in loading direction and crossways direction is 8 fiber yarns and 24 fiber yarns, respectively.

**Figure 4:**
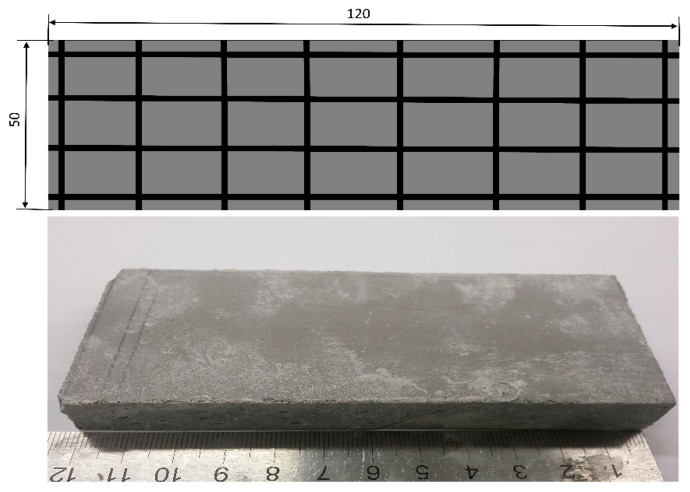
The sample size of the TRG specimens for the Charpy impact test (unit: mm).

The TRG specimens with approximate dimensions of 15 × 50 × 120 mm^3^, which were cut off from the 15 × 100 × 400 mm^3^ specimens, were used to test the Charpy impact resistance. Figure 4 shows the number of fiber yarns arranged in the samples and the sample size. Note that in the case of the Charpy impact test, several additional specimens reinforced with 2–3 textile layers were fabricated. The purpose of this is to evaluate the Charpy impact resistance of the TRG specimens with multiple layers. The arrangement of the multiple layers in the samples has been detailed in the previous work [44].

### 2.3. Test Methods

**Figure 5:**
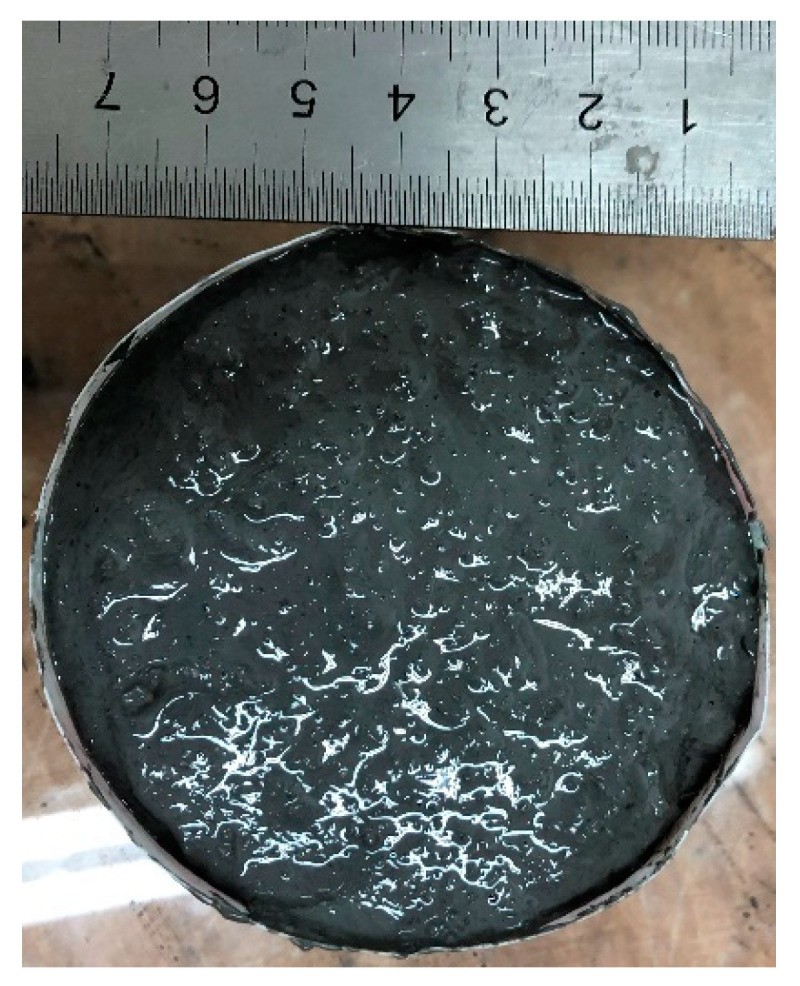
Geomortar inside the plastic cup.

The observation of the self-flowability of the fresh geomortar containing various dosages of CBFs can be determined by the simple method, as shown in Figure 5. The fresh geomortar, after finishing the mixing, was poured into a plastic cup with a top diameter of about 75 mm and bottom diameter of about 85 mm and an approximate height of 55 mm. The plastic cup was then lifted away from the geomortar, and the observation of the self-flowability of the geomortar was recorded over 60 s.

The static flexural behavior of the TRG composites was applied by the four-point bending test. A detailed description of the specimen arrangement and testing process was shown in the previous publication [43]. The testing machine with a load cell capacity of 100 kN (FP Lab Test II, from LABORTECH s.r.o, Opava, Czech Republic), located at the Technical University of Liberec Laboratory, with an applied load under displacement control at a loading rate of 4 mm∙min^−1^, was used. The test was repeated for the three specimens, and a mean value of measurements was obtained. The four-point flexural strength where the loading span is 1/3 of the support span (rectangular cross-section) was calculated as per Equation (1) [19]:

*σ* = *Fl*/(*bh*^2^) (1)

where *σ* is the four-point flexural strength in MPa; *F* is the load at a given point on the load-displacement curve in N; *b* is the width of the tested sample in mm; *h* is the thickness of the sample in mm; *l* is the support span in mm.

The dynamic flexural behavior of the TRG specimens was determined by the Charpy impact test. The Charpy test machine was modified to fit a desired size of 15 × 50 × 120 mm^3^, and to ensure the loading span of 100 mm of the tested specimens. A Charpy impact tester with an 18 kg pendulum hammer was employed to determine the impact strength. The six specimens for each recipe were applied in the test, and an average of measurements was recorded. The impact flexural strength can be calculated as per Equation (2) [45]:

σ_i_ = E/A (2)

where σ_i_ is the impact strength in MPa; E is the impact energy required to break the tested sample in Joule (J); A is the cross-section area of the tested sample in m^2^.

## 3. Results

### 3.1. Self-Flowability of the Fresh Geomortar Matrix

**Figure 6:**
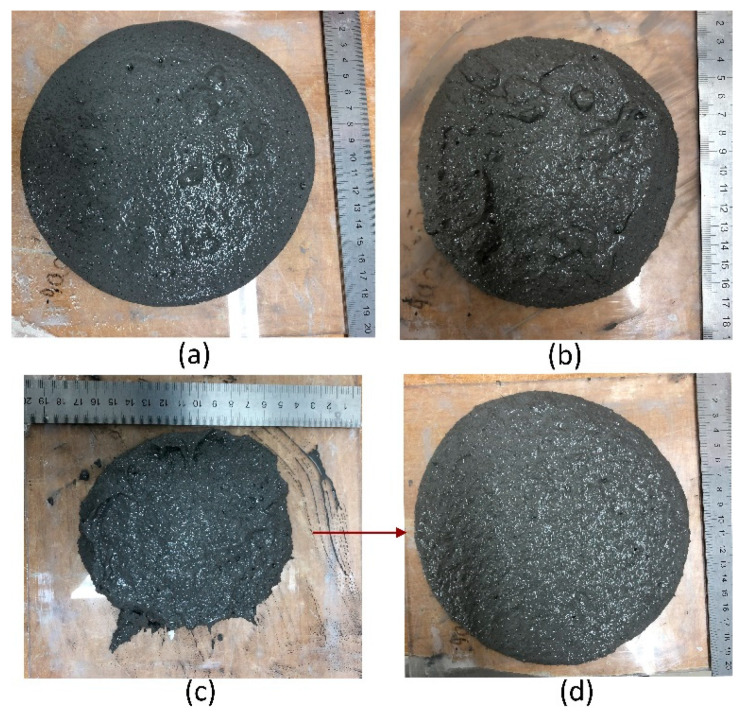
Self-flowability of the geomortar containing various dosages of CBFs: (**a**) geomortar without CBF adding and without vibrating; (**b**) geomortar with 0.25% 6mm CBFs without vibrating; (**c**) geomortar with 0.5% 6 mm CBFs without vibrating; (**d**) geomortar with 0.5% 6 mm CBFs after vibrating.

The observation of the self-flowability of the fresh geomortar containing various dosages of CBFs is shown in Figure 6. The results showed that with an increase in fiber concentration or fiber length, CBFs significantly hindered the self-flow, resulting in the high viscosity of geomortar. Figure 6a shows the workability of the geomortar without adding CBFs. The very good self-flow of this mortar is clearly seen, and it has achieved the highest flowability as compared to the rest of the mixtures in the experiment. The geomortar containing 0.25% of 6-mm CBFs has shown a significant decrease in flowability (Figure 6b), as compared to the geomortar in Figure 6a. In the mixture with a CBF dose of 0.5% or more, the geomortar was almost incapable of flowing by itself (Figure 6c). Thus, the specimens produced by these mixes must be vibrated to ensure the homogenous consistency of the geomortar in the molds during sample preparation (Figure 6d). A similar behavior of the poor flowability was observed in the mixes containing 12 mm CBFs and 24 mm CBFs. However, for these two types of CBFs, the mixture only with a dose of 0.25% had a poor flow similar to the mixture containing 0.5% 6-mm CBFs. This means that CBFs with longer fiber lengths reduced mortar flow more. Moreover, it is also worth noticing that while the 6 mm CBFs are dispersed easily in the mixture, the mixing difficulty has occurred by using 12 mm CBFs and 24 mm CBFs due to the fiber entanglement. This leads to the presence of several clusters of fibers in the mixture, as the concentration of CBFs starts at 0.5%.

### 3.2. Flexural Load-Displacement Response of the TRG Composites and Their Flexural Properties

**Figure 7:**
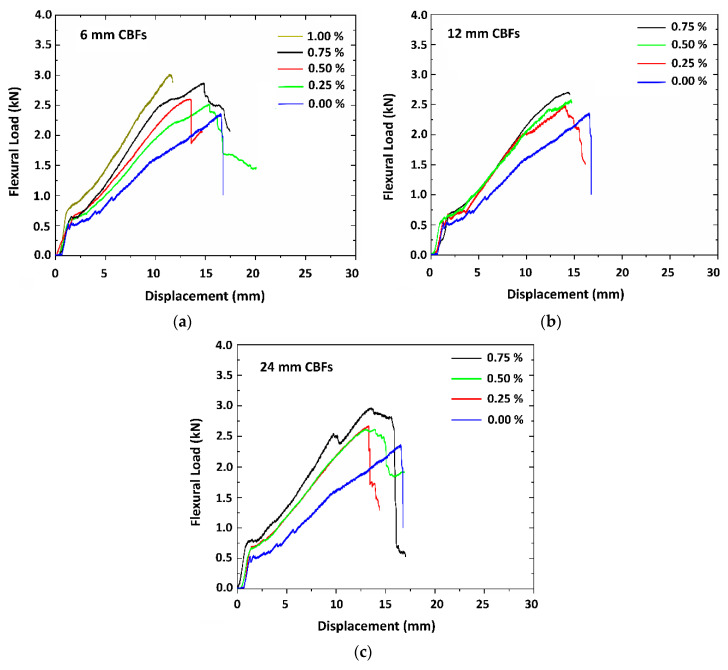
Average flexural load-displacement curves of the TRG composites: (**a**) TRG specimens with the addition of the 6 mm CBFs; (**b**) TRG specimens with the addition of the 12 mm CBFs; (**c**) TRG specimens with the addition of the 24 mm CBFs.

Figure 7a–c shows the response of the typical load-displacement curves obtained from four-point bending tests of the TRG specimens with the various dosages of CBF addition. It is apparent from the diagrams that CBF addition was efficient in increasing the load-bearing capacity of the hybrid TRGs compared to the reference TRGs. The load-displacement curves also showed that CBF reinforcement helps hybrid TRGs to become stiffer and stronger, as seen through the higher slope, and leads to less oscillation of curves and less displacement as compared to the reference TRGs. Although there is some variation in the behavior of the individual curves, the general load-displacement response is roughly similar for all the composites. After reaching the maximum load, most specimens are virtually incapable of carrying the load further. A significant flattening of the load-displacement curves around the point of maximum load associated with inelastic deformation is displayed for all the hybrid TRGs, except to the reference TRGs. In some cases, the bending test was ended immediately after reaching the maximum load. In other cases, the bending load decreased slowly in a short duration, increasing the displacement.

**Figure 8:**
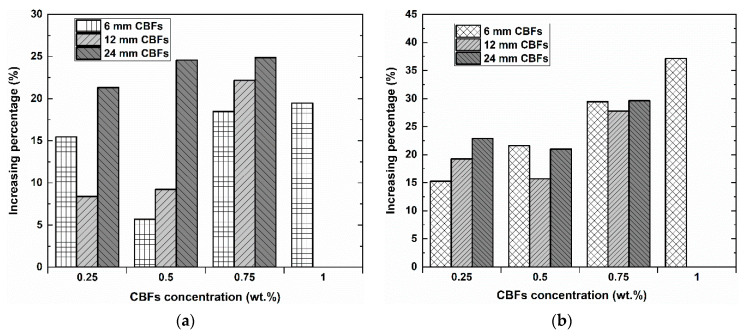

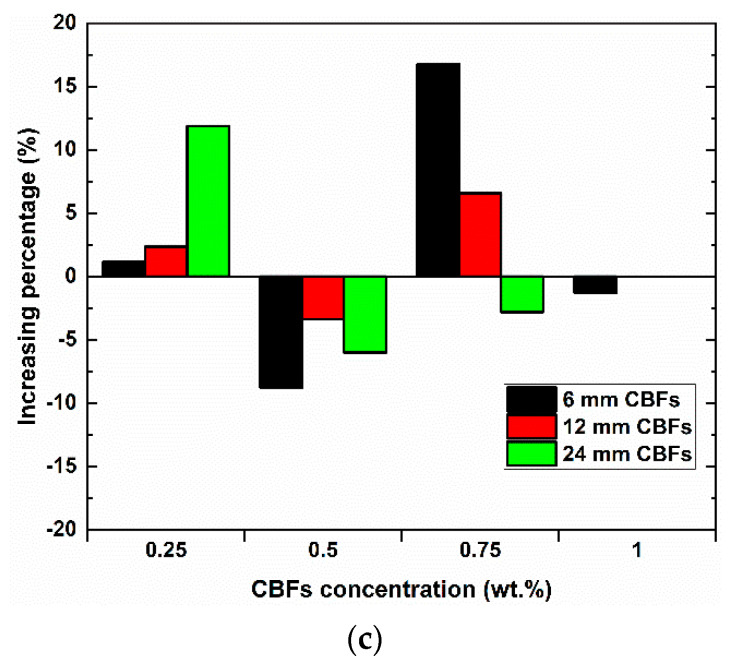
Comparison of the mechanical strength between the TRG specimens containing the CBF addition and the reference TRGs: (**a**) increasing percentage of the first-crack strength; (**b**) increasing percentage of the ultimate strength; (**c**) increasing percentage of the flexural toughness.

Table 4 provides the information about the results of the flexural test of all the TRG specimens, including first-crack load, ultimate load, first-crack strength, ultimate strength, displacement, flexural toughness, and the number of cracks, whereas Figure 8 shows a comparison of the difference of the increasing percentage of the mechanical strength between the hybrid TRGs and the reference TRGs. As Table 4 and Figure 8 show, CBF reinforcement distributed its usefulness into TRGs early in the initial stage, as seen through the first-crack bending strength of all the hybrid TRG specimens, which was always higher than that of the reference TRGs. This usefulness continues to remain constant until the hybrid TRGs reach the maximum load capacity. The reference TRGs have the average ultimate flexural strength of 30.14 MPa. The highest value of ultimate bending strength was 41.33 MPa for the hybrid TRGs with the 1% 6 mm CBF addition. The next good values of the bending strength were 39.07 MPa and 38.51 MPa for the hybrid TRGs with 0.75% adding of the 24 mm CBFs and the 12 mm CBFs, respectively, considering the higher variability of measured values of the strength. It can be seen that for all three different fiber lengths of CBFs, the specimens obtained the highest bending strength with the highest CBF concentration. However, keep in mind the fact that the higher the CBF loading, the harder it is for the fresh geomortar to be mixed homogenously, and this results in the marked occurrence of the fiber clusters. This is reason why the authors did not want to increase the CBF concentration over 0.75%. The increasing percentage in strength of the hybrid TRGs compared to reference TRGs ranges from about 5 to 25% at the first-crack load and from about 15 to 37% at the peak load (Figure 8a–b). This confirmed that CBFs have improved the ultimate strength better than the first-crack strength in TRGs. In other words, the effect of CBFs on the bending toughness of the specimens is unclear (Figure 8c). From the experimental results, it can be seen that the specimens with higher flexural strength do not always indicate a higher bending toughness than those with lower flexural strength and vice versa. The reason for this is that the toughness value of the TRG specimens in this work was calculated only at the point of ultimate load, and hence this value depends simultaneously on both the load-bearing capacity and displacement of each TRG. For some TRG specimens with an increase in flexural strength, on the other hand, there was a significant reduction in displacement, leading to the lower toughness value, as compared to the reference TRGs.

### 3.3. Failure Modes

**Figure 9:**
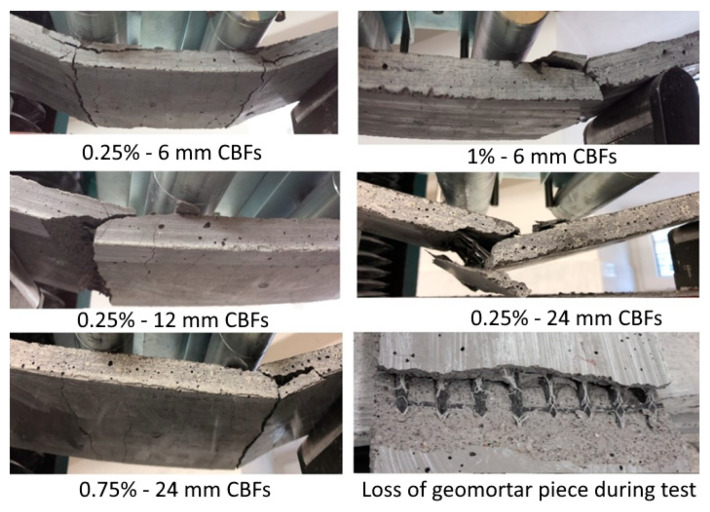
Typical failure modes of the hybrid TRGs regarding different fiber lengths and various dosages of CBFs.

Photographs of typical failure observations for these TRG specimens are shown in Figure 9. The results from Figure 9 clearly show that all the composites failed in a catastrophic manner, which was demonstrated by the process of debonding or simultaneous debonding and collapse after reaching the maximum load-bearing capacity. In some cases, the specimens failed due to the occurrence of debonding along with the matrix-textile interface. In other cases when specimens failed, the geomortar pieces were broken down, but debonding along the matrix-textile interface did not occur. Finally, these specimens broke down due to the collapse of the matrix. The failure modes indicated that although using CBFs could enhance the flexural strength of the hybrid TRGs, there was no improvement in failure modes as compared to the reference TRGs. It can be confirmed that both fiber lengths and dosages used in this research do not have any appreciable difference in the failure modes of the TRG specimens.

### 3.4. Charpy Impact Performance of the TRG Composites

**Figure 10:**
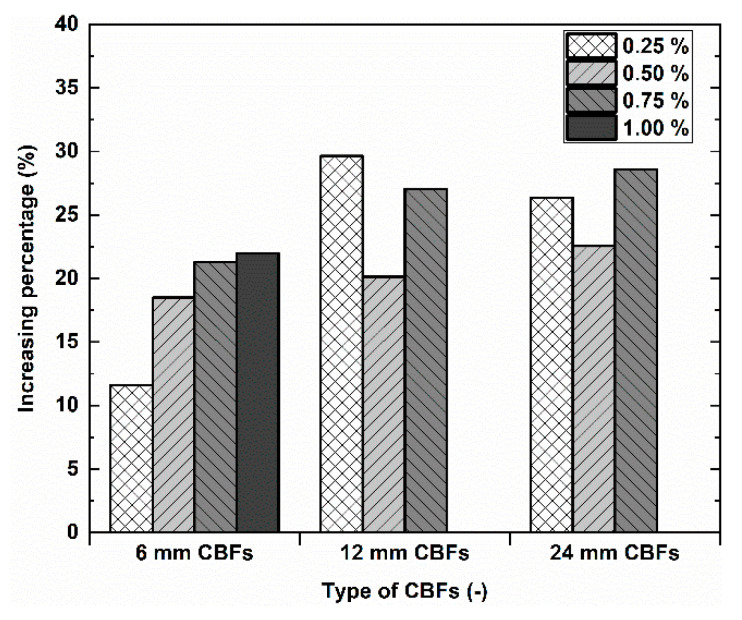
Comparison of the impact strength between the TRG specimens containing CBF addition and reference TRG specimens.

**Figure 11:**
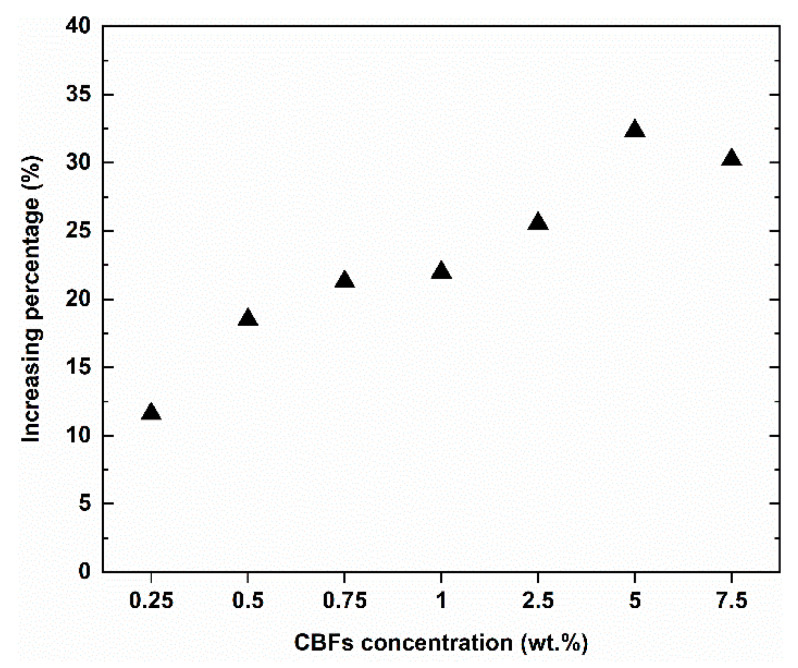
Comparison of the impact strength between the TRG specimens with the 6 mm CBF addition and the reference TRGs.

The experimental results of the Charpy impact tests for the TRG specimens with CBF addition and reference TRGs were reported in Table 5, Figure 10 and Figure 11. It is noted that the 6 mm CBF concentrations at high dose levels (2.5%, 5%, 7.5%) described in previous publication [44] were also applied to evaluate the Charpy impact strength (Table 5). The reference TRGs show the lowest average impact strength, with a value of 60.86 kJ/m^2^. After adding varied dosages of CBFs to TRG, the average impact strength ranged from 67.92 kJ/m^2^ to 80.55 kJ/m^2^, which was increased by 11.66 to 32.35%. This finding confirmed that CBFs also help to improve the mechanical strength of the TRG specimens significantly under dynamic flexural load. However, an observation in the value of the standard deviation also indicated confusing variability. As shown in Table 5, at the low dose level, TRGs with the addition of a dosage of 0.25% and 0.75% for all three CBFs kinds showed a smaller standard deviation than those of 0.5%. Moreover, the resulting strength of TRGs for each fiber type at each dosage was generally unpredictable. For example, while TRGs with the 0.25% 12 mm CBF addition showed the highest impact strength as compared to those at the other two dosage percentages, the highest impact strength for TRGs with the 24 mm CBF addition was achieved at a dosage of 0.75%. This result can be attributed to the fabrication process. Since all the TRG specimens have been fabricated according to the same technique and from the same materials, this behavior can be attributed to the presence of several clusters of fibers in the mixture, which resulted in a non-homogenous structure of the hardened TRGs. In other words, from the results in Table 5 and Figure 10, it can be observed that TRGs with 6 mm CBFs show a lower impact strength value compared to those with the other two CBF types, considering a low dose level. This result is inconsistent with the results of these TRGs in the static flexural loading test (four-point bending test), where the highest strength value was achieved for TRGs with the 1% 6 mm CBF addition. This finding shows that the failure mechanism of TRGs under static and impact loading is different. Figure 10 also shows that the increasing percentage of the impact strength of TRGs with the CBF addition ranges from about 12 to 30%, as compared to the reference TRGs.

The average impact strength of TRGs with varied addition of the 6 mm CBFs is compared in Table 5 and Figure 11. By increasing the CBF content, TRGs showed an increased trend in strength. Furthermore, adding CBFs at a high dose level to the geomortar appears to have caused TRGs to achieve a more homogeneous strength within each recipe due to a smaller value of standard deviation, as compared to that of TRGs with the CBF addition at the low dose levels. This can be attributed to the CBF amount added to the geomortar. If the amount of CBFs added still ensures the good workability of fresh mortar and the mechanical strength of hardened mortar, the more the CBFs added, the more homogeneous mortar.

**Figure 12:**
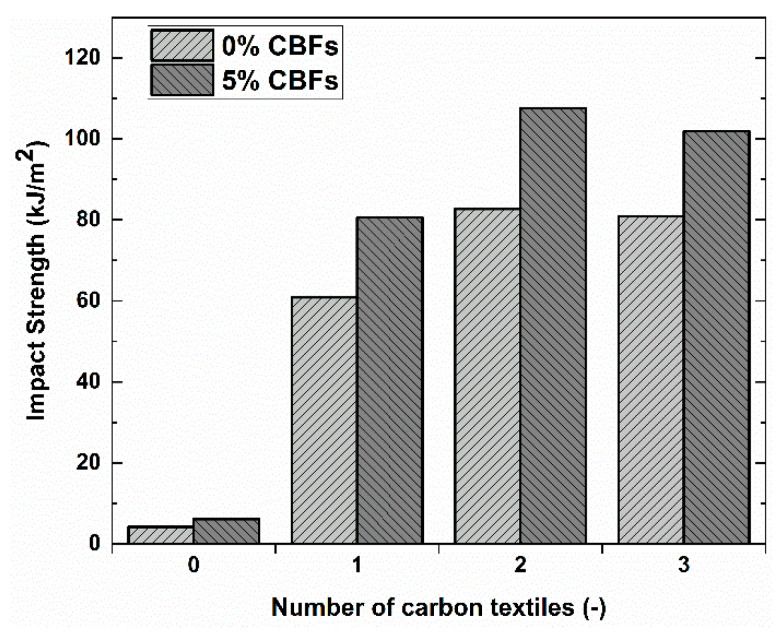
The result of the bending impact strength of TRGs reinforced with 1–3 textile layers.

The experimental results of the Charpy impact test for TRGs reinforced with 1–3 layers of the C-10 × 15 textile were reported in Table 6 and Figure 12. Two mortar types were used, one without the CBF addition and the other with 5% CBF addition. From Table 6 we can see that the non-textile specimens without the CBF addition achieved the lowest impact strength, of 4.16 kJ/m^2^, which showed a ~1.47 times lower average strength as compared to those with 5% CBF addition (6.1 kJ/m^2^). The CBF reinforcement also enhanced the energy absorption capacity of TRGs considering the same reinforcement ratio, when comparing to the reference TRGs. The 5% CBF TRGs reinforced with 1–3 layers increased by 1.32 times, 1.30 times, and 1.24 times, respectively, as compared to the reference TRGs. It is also observed that the higher average strength is accompanied by a higher standard deviation of measured strength. This problem of standard deviation could be attributed to the fact that the higher the reinforcement ratio, the higher the probability of strength difference between the specimens due to the damage of fiber yarns in each textile layer, which occurred in its manufacture. The experimental results showed that a clear improvement for TRGs was observed with an increasing reinforcement ratio. However, it can be seen that TRGs reinforced with three layers does not show a higher average strength value than TRGs reinforced with two layers, even smaller (Figure 12). This phenomenon can be attributed to the anchoring capacity of textile reinforcement in the geomortar under the dynamic loading test. For three-layer TRGs, because the geomortar cover thickness between two adjacent textile layers was 2 mm, as described in reference [44], this coating thickness may not be strong enough to anchor the fiber yarns of textile under the impact load.

**Figure 13:**
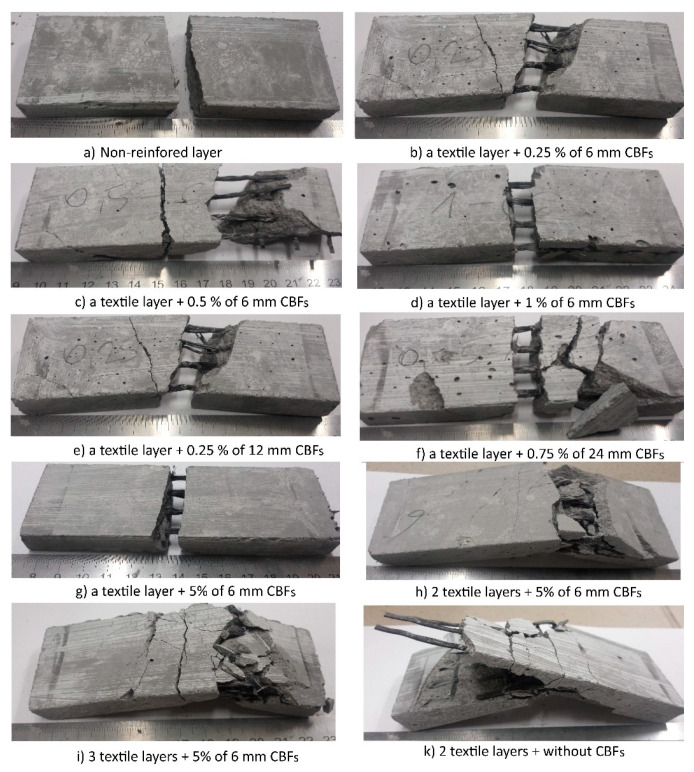
Typical failure modes of the TRG specimens after the Charpy impact test.

Figure 13 shows the typical failure modes of the TRG specimens after finishing the Charpy impact test. The dominating failure mode for these TRGs was the slip of fiber yarns within the geomortar matrix along with a partial damage of the multifilament at the outer layer; no bundle of fibers was broken. Moreover, from Figure 13 it is observed that the one-layer TRGs have shown a different failure manner, as compared to those with 2–3 layers. The one-layer TRGs were separated into two main parts with a separating distance of ~ 10–15 mm caused by the slipping of fiber bundles. In some cases, the specimens failed disastrously; in other cases, specimens failed smoothly. TRGs with 2–3 layers, in contrast, lost their structural geometry and integrity upon reaching the impact energy capacity, which cannot return to their original shape after finishing the test. The TRG specimens bend around the head of the hammer but do not separate due to the flexibility and toughness of fiber yarns. This evidence showed that one reinforcing layer can receive the collision energy and transmit it well, while a higher reinforcement ratio of the carbon textile applied in TRGs makes its usefulness not fully utilized.

## 4. Comparison with our Previous Research and General Discussion

In this part, the authors would like to give an overall assessment of the effectiveness of the CBFs used as additional reinforcement for carbon textile-reinforced geomortar. In the current work, CBFs with varied loadings (0.25%, 0.5%, 0.75%, 1%) added to the geomortar will be marked as low dose level, whereas the high dose level used much higher CBF loadings, including 2.5%, 5%, 7.5%, and has been reported in previous publication [44]. A comparison and discussion of the efficacy of CBFs on the flexural behavior of carbon textile-reinforced geomortar are presented in the lines below.

In the previous research [44], the authors applied very high doses of CBFs to evaluate their effectiveness on the flexural behavior of TRGs. The disadvantage of using CBFs at a high dose level is the mixing process. It requires that the mixer operate at high capacity, and the mixing takes a long time for the mixture to be homogenized. Therefore, the original fiber lengths of CBFs were completely chopped to a smaller size, that cannot be controlled. Therefore, the mechanical properties of these TRGs are influenced only by the CBF concentration. The outstanding advantages can be achieved from the high CBF doses, such as good flowability, the absence of fiber clusters, homogenous fresh mix, and high geomortar strength. Using CBFs at a low dose level in this work, in contrast, brought the following consequences: ease of mixing process, fibers entangled on the mixing paddle, causing the appearance of fiber clusters, as well as heterogeneous mixture, low flowability, maintained fiber length, and low geomortar strength. Therefore, the mechanical properties of these TRGs are influenced by both CBF concentration and fiber length.

As the results of the four-point bending test of the TRG specimens show in this work, using CBFs at a low dose level can help the TRG specimens to achieve flexural strength, which has a similar value to that of the TRG specimens containing CBFs at a high dose level (seen in reference [44]). This means that both dose levels contribute to enhancing the load-bearing capacity of the TRG specimens. The high CBF dose enhances the flexural strength through fiber concentration while the low CBF dose takes into account fiber concentration and length. However, the TRG specimens using high CBF doses exhibited a very positive manner of failure as compared to those using low CBF doses. Despite using the varying dosages (from 0.25 to 1%), the TRG specimens at a low dose level always fail in catastrophic mode, which is demonstrated by the process of debonding or simultaneous debonding and collapse after reaching the maximum load-bearing capacity (seen in Figure 9). This behavior indicated that CBFs at a low dose level almost lose their usefulness in TRGs, or they cannot help TRGs to maintain its load-bearing capacity after the TRG specimens reach the maximum load-bearing capacity. Using CBFs at a high dose level, on the other hand, resulted in pure flexural failure without the debonding stage and sudden collapse. After reaching the maximum load-bearing capacity, the specimens fail smoothly, characterized by a gradual decrease of the curves in load; they were reduced to roughly 0.6 times their maximum load capacity. These TRGs are then able to maintain the load-bearing capacity by increasing the displacement (Figure 7 in reference [44]). The failure of these specimens is thought to have been caused by a progressive slippage of fiber yarns in the geomatrix (Figure 9 in reference [44]). From this point of view, we can confirm that CBFs will have a very positive effect on the flexural behavior of the TRG composites if they are considered to apply in an appropriate dosage. The results obtained in the Charpy impact test have also shown an interesting finding. The three-layer TRG specimens do not display a higher value of the impact strength than those reinforced with two layers (Figure 12). However, under the four-point bending test presented in reference [44], TRGs with three reinforcing layers showed a much higher flexural strength than those with two reinforcing layers (seen Figure 5a in [44]). This finding showed that there is a significant difference in anchoring capacity of textile reinforcement in the geomortar between static and dynamic loads. Compared to the static bending test, the dynamic bending test requires a stronger geomortar cover layer between textile layers. The authors recommend that future works find out the optimal geomortar cover layer of TRGs reinforced with multiple layers of carbon textile in order to make the most of its usefulness under a dynamic load.

## 5. Conclusions

The geopolymer based hybrid composites containing carbon textile and chopped basalt fibers have been experimentally investigated. The flexural properties of TRGs, including static bending strength and dynamic bending strength, were measured and evaluated. According to the mixing method applied, the fresh geomortar showed a significant reduction in flowability. Therefore, the specimens should be vibrated carefully during manufacturing to ensure the dense packing of the geomortar in the molds. The 6 mm CBFs did not cause the clumping in fresh geomortar. A mixture with the addition of 12 mm CBFs and 24 mm CBFs, in contrast, resulted in the appearance of fiber clusters in the geomortar. This could be one of the main reasons why there is a high variability of measured values of the specimen’s strength. As compared to results in the previously published work [44], adding CBFs to geomortar in this work only improves the flexural strength of the hybrid TRGs, and there is no improvement in the failure mode. Their failure is similar to that of the reference TRGs due to fiber debonding or, simultaneously, fiber debonding and sudden collapse. CBFs also enhanced the impact resistance of the TRGs. The impact strength of the one-layer TRGs is much higher than that of the non-textile layer specimens. The strength of the two-layer TRGs keeps increasing as compared to that of the one-layer TRGs. However, the three-layer TRGs showed a decrease in strength compared to the two-layer TRGs. In short, this study provides information on utilizing CBFs for improving the mechanical properties of TRGs. It confirmed that CBFs will have a very positive effect on the flexural behavior of TRGs in the case of an appropriate dosage selection. A length of 6 mm appears to be a reasonable choice compared to two other lengths of CBFs.

## Figures and Tables

**Table 1:** Material characteristics of textile reinforcement provided by the manufacturer.

Fiber Type	Carbon HTC 10/15–40
Fiber density	1.77 g/cm^3^
Number of threads/m	78 (lengthways); 55 (crossways)
Weight	350 g/m^2^
Tex	3200 g/km
Stitch spacing	10 × 15 mm^2^ (center to center distance)
Tensile strength	2551 MPa (lengthways); 2847 MPa (crossways)
Young’s Modulus	228 GPa (lengthways) 252 GPa (crossways)
Elongation	1.17% (lengthways) 1.24% (crossways)

**Table 2:** Mix proportions of the geomortar matrix by weight ratio.

Geocement	Activator	Rough Sand	Micro-Milled Sand	Silica Fume	CBFs
1	0.8	1.5	0.2	0.1	0, 0.25, 0.5, 0.75, 1.0

CBFs dosage is calculated by mass percentage of the geopaste.

**Table 3:** Mechanical properties of the geomortar at the age of 28 days.

Type (-)	Fiber Dosage (wt.%)	Flexural Strength (MPa)	Compressive Strength (MPa)
	0.00	11.78 ± 0.48	79.56 ± 3.32
6 mm CBFs	0.25	11.96 ± 0.27	80.05 ± 5.67
	0.50	13.43 ± 0.85	77.01 ± 3.13
	0.75	12.28 ± 0.33	76.56 ± 4.68
	1.00	13.33 ± 0.56	75.24 ± 4.01
12 mm CBFs	0.25	12.96 ± 0.50	77.18 ± 1.27
	0.50	11.85 ± 0.13	83.37 ± 1.31
	0.75	12.65 ± 0.15	83.77 ± 1.06
24 mm CBFs	0.25	12.25 ± 0.78	81.82 ± 1.08
	0.50	13.05 ± 0.65	82.47 ± 3.10
	0.75	14.10 ± 0.77	78.51 ± 2.25

**Table 4:** Mechanical properties of the TRG specimens without and with the addition of CBFs.

	Value	F_1_ (kN)	F_2_ (kN)	σ_1_ (MPa)	σ_2_ (MPa)	y (mm)	Toughness (kN.mm)	Cr. (-)
Sample								
	0.00%	0.53 ± 0.09	2.26 ± 0.71	7.04 ± 1.15	30.14 ± 1.29	16.59 ± 2.65	23.43 ± 2.57	8.66
**6 mm CBFs**	0.25%	0.61 ± 0.04	2.69 ± 0.44	8.13 ± 0.50	34.74 ± 1.72	14.47 ± 1.27	23.71 ± 1.49	11.33
	0.50%	0.56 ± 0.04	2.75 ± 0.28	7.44 ± 0.49	36.65 ± 3.77	13.73 ± 1.19	21.37 ± 1.78	12.33
	0.75%	0.63± 0.02	2.93± 0.18	8.34 ± 0.24	39.01 ± 2.36	15.03 ± 1.08	27.36 ± 3.37	11.67
	1.00%	0.63 ± 0.04	3.10 ± 0.11	8.41 ± 0.52	41.33 ± 1.41	12.83 ± 2.30	23.13 ± 5.30	13.33
**12 mm CBFs**	0.25%	0.57 ± 0.06	2.69 ± 0.18	7.63 ±0.85	35.94 ± 2.45	15.65 ± 2.51	23.98 ± 3.86	12.00
	0.50%	0.58 ± 0.09	2.57 ± 0.12	7.69 ± 1.33	34.87 ± 1.65	14.68 ± 1.61	22.63 ± 2.49	12.00
	0.75%	0.64 ± 0.04	2.81 ± 0.24	8.60 ± 0.50	38.51± 3.24	14.72 ± 1.34	24.97± 4.76	12.67
**24 mm CBFs**	0.25%	0.64 ± 0.02	2.77 ± 0.22	8.54 ± 0.25	37.04 ± 2.93	15.28 ± 1.91	26.21± 5.40	13.33
	0.50%	0.65 ± 0.02	2.74 ± 0.29	8.77 ± 0.25	36.47 ± 3.88	13.65 ± 1.18	22.02 ± 4.13	11.67
	0.75%	0.66 ± 0.04	2.93 ± 0.27	8.79 ± 0.55	39.07 ± 3.57	13.41 ± 1.43	22.77 ± 4.35	13.67

Symbols, such as F_1,_ F_2,_ σ_1_, σ_2_, y, and Cr., indicate the first-crack load, ultimate load, first-crack strength, ultimate strength, displacement, and number of cracks, respectively.

**Table 5:** The result of the Charpy impact test of the TRG specimens.

The TRG Specimens with Varied Additions of CBFs			
Type	CBFs [wt.%]	K [J]	KC [kJ/m^2^]
Non CBFs	0.00	46.07 ± 7.27	60.86 ± 09.27
6 mm CBFs	0.25	51.79 ± 9.10	67.92 ± 12.32
	0.50	54.67 ± 10.33	72.12 ± 14.04
	0.75	56.63 ± 8.94	73.81 ± 12.54
	1.00	56.13 ± 6.22	74.23 ± 08.16
	2.50	58.38 ± 5.98	76.41 ± 06.71
	5.00	61.46 ± 6.42	80.55 ± 08.74
	7.50	61.17 ± 8.32	79.26 ± 09.10
12 mm CBFs	0.25	59.49 ± 7.04	78.90 ± 08.63
	0.50	56.17 ± 9.80	73.11 ± 12.66
	0.75	58.92 ± 4.07	77.32 ± 08.46
24 mm CBFs	0.25	58.96 ± 6.90	76.90 ± 11.10
	0.50	56.50 ± 10.07	74.59 ± 14.07
	0.75	59.13 ± 7.64	78.26 ± 08.53

**Table 6:** The results of the Charpy impact test of geocomposites reinforced with carbon textile.

	Value		Geomortar without CBF Addition		Geomortar with 5% CBF Addition	
Sample		No. Layer	K [J]	KC [kJ/m^2^]	K [J]	KC [kJ/m^2^]
Non-reinforced sample		0L	3.14 ± 1.04	04.16 ± 1.35	4.63 ± 1.34	06.10 ± 1.64
TRGs		1L	46.07 ± 7.27	60.86 ± 07.27	61.46 ± 6.42	80.55 ± 8.74
		2L	62.42 ± 13.23	82.67 ± 13.84	82.63 ±13.23	107.55 ± 16.61
		3L	60.59 ± 14.93	81.85 ± 16.58	77.96 ±14.93	101.88 ± 18.69

## Data Availability

The data presented in this study are available on request from the corresponding author.

## References

[1] Cevallos O., Olivito R. (2015). Effects of fabric parameters on the tensile behaviour of sustainable cementitious composites. Compos. Part B Eng..

[2] D’Antino T., Papanicolaou C. (2017). Mechanical characterization of textile reinforced inorganic-matrix composites. Compos. Part B Eng..

[3] Lignola G.P., Caggegi C., Ceroni F., de Santis S., Krajewski P., Lourenço P.B., Morganti M., Papanicolaou C., Pellegrino C., Prota A. (2017). Performance assessment of basalt FRCM for retrofit applications on masonry. Compos. Part B. Eng..

[4] Ferrara G., Coppola B., Di Maio L., Incarnato L., Martinelli E. (2019). Tensile strength of flax fabrics to be used as reinforcement in cement-based composites: Experimental tests under different environmental exposures. Compos. Part B Eng..

[5] Colombo I.G., Colombo M., Di Prisco M. (2015). Bending behaviour of Textile Reinforced Concrete sandwich beams. Constr. Build. Mater..

[6] Mechtcherine V. (2013). Novel cement-based composites for the strengthening and repair of concrete structures. Constr. Build. Mater..

[7] Portal N.W., Flansbjer M., Zandi K., Wlasak L., Malaga K. (2017). Bending behaviour of novel Textile Reinforced Concrete-foamed concrete (TRC-FC) sandwich elements. Compos. Struct..

[8] Dey V., Zani G., Colombo M., Di Prisco M., Mobasher B. (2015). Flexural impact response of textile-reinforced aerated concrete sandwich panels. Mater. Des..

[9] Yin S., Wang B., Wang F., Xu S. (2017). Bond investigation of hybrid textile with self-compacting fine-grain concrete. J. Ind. Text..

[10] Shiping Y.I.N., Shilang X.U., Hedong L.I. (2013). Improved Mechanical Properties of Textile Reinforced Concrete Thin Plate. J. Wuhan Univ. Technol. Mat. Sci. Edit..

[11] Li Q., Xu S. (2010). Experimental Research on Mechanical Performance of Hybrid Fiber Reinforced Cementitious Composites with Polyvinyl Alcohol Short Fiber and Carbon Textile. J. Compos. Mater..

[12] Dvorkin D., Peled A. (2016). Cement and concrete research effect of reinforcement with carbon fabrics impregnated with nanoparticles on the tensile behavior of cement-based composites. Cem. Concr. Res..

[13] Du Y., Zhang X., Zhou F., Zhu D., Zhang M., Pan W. (2018). Flexural behavior of basalt textile-reinforced concrete. Constr. Build. Mater..

[14] Ding Y., Wang Q., Pacheco-Torgal F., Zhang Y. (2020). Hybrid effect of basalt fiber textile and macro polypropylene fiber on flexural load-bearing capacity and toughness of two-way concrete slabs. Constr. Build. Mater..

[15] Zhu D., Liu S., Yao Y., Li G., Du Y., Shi C. (2019). Effects of short fiber and pre-tension on the tensile behavior of basalt textile reinforced concrete. Cem. Concr. Compos..

[16] Liu S., Zhu D., Li G., Yao Y., Ou Y., Shi C., Du Y. (2018). Flexural response of basalt textile reinforced concrete with pre-tension and short fibers under low-velocity impact loads. Constr. Build. Mater..

[17] Du Y., Zhang M., Zhou F., Zhu D. (2017). Experimental study on basalt textile reinforced concrete under uniaxial tensile loading. Constr. Build. Mater..

[18] Barhum R., Mechtcherine V. (2013). Influence of short dispersed and short integral glass fibres on the mechanical behaviour of textile-reinforced concrete. Mater. Struct..

[19] Du Y., Zhang X., Liu L., Zhou F., Zhu D., Pan W. (2018). Flexural Behaviour of Carbon Textile-Reinforced Concrete with Prestress and Steel Fibres. Polymers.

[20] Pakravan H.R., Jamshidi M., Rezaei H. (2016). Effect of textile surface treatment on the flexural properties of cementitious composites. J. Ind. Text..

[21] Peled A., Zaguri E., Marom G. (2008). Bonding characteristics of multifilament polymer yarns and cement matrices. Compos. Part A.

[22] Davidovits J. (1989). Geopolymers and geopolymeric materials. J. Therm. Anal..

[23] Duxson P., Mallicoat S., Lukey G., Kriven W., van Deventer J. (2007). The effect of alkali and Si/Al ratio on the development of mechanical properties of metakaolin-based geopolymers. Colloids Surf. A Physicochem. Eng. Asp..

[24] Da Silva Rocha T., Dias D.P., França F.C.C., de Salles Guerra R.R., da Costa de Oliveira Marques L.R. (2018). Metakaolin-based geopolymer mortars with different alkaline activators. Constr. Build. Mater..

[25] Yu X., Chen L., Komarneni S., Hui C. (2016). Fly ash-based geopolymer: Clean production, properties and applications. J. Clean. Prod..

[26] Abdalqader A.F., Jin F., Al-Tabbaa A. (2016). Development of greener alkali-activated cement: Utilisation of sodium carbonate for activating slag and fly ash mixtures. J. Clean. Prod..

[27] Tennakoon C., Shayan A., Sanjayan J.G., Xu A. (2017). Chloride ingress and steel corrosion in geopolymer concrete based on long term tests. Mater. Des..

[28] Singh B., Rahman M., Paswan R., Bhattacharyya S. (2016). Effect of activator concentration on the strength, ITZ and drying shrinkage of fly ash/slag geopolymer concrete. Constr. Build. Mater..

[29] Nazari A., Bagheri A., Sanjayan J.G., Dao M., Mallawa C., Zannis P., Zumbo S. (2019). Thermal shock reactions of Ordinary Portland cement and geopolymer concrete: Microstructural and mechanical investigation. Constr. Build. Mater..

[30] Hussin M.W., Bhutta M.A.R., Azreen M., Ramadhansyah P.J., Mirza J. (2015). Performance of blended ash geopolymer concrete at elevated temperatures. Mater. Struct..

[31] Menna C., Asprone D., Ferone C., Colangelo F., Balsamo A., Prota A., Cioffi R., Manfredi G. (2013). Use of geopolymers for composite external reinforcement of RC members. Compos. Part B Eng..

[32] Hung T.D., Louda P., Kroisova D., Bortnovsky O., Xiem N.T., Těšinova P. (2012). New Generation of Geopolymer Composite for Fire-Resistance. Advances in Composite Materials—Analysis of Natural and Man-Made Materials.

[33] Khalid H.R., Ha S., Park S.M., Kim G., Lee H. (2015). Interfacial bond behavior of FRP fabrics bonded to fiber-reinforced geopolymer mortar. Compos. Struct..

[34] Samal S., Marvalová B., Petríková I., Vallons K.A.M., Lomov S.V., Rahier H. (2016). Impact and post impact behavior of fabric reinforced geopolymer composite. Constr. Build. Mater..

[35] Rill E., Lowry D.R., Kriven W.M. (2010). Properties of Basalt Fiber Reinforced Geopolymer Composites. Strategic Materials and Computational Design—A Collection of Papers Presented at the 34th International Conference on Advanced Ceramics and Composites.

[36] Ribero D., Kriven W.M. (2016). Properties of Geopolymer Composites Reinforced with Basalt Chopped Strand Mat or Woven Fabric. J. Am. Ceram. Soc..

[37] Shaikh F., Haque S. (2018). Behaviour of Carbon and Basalt Fibres Reinforced Fly Ash Geopolymer at Elevated Temperatures. Int. J. Concr. Struct. Mater..

[38] Zhang H.Y., Yan J., Kodur V., Cao L. (2019). Mechanical behavior of concrete beams shear strengthened with textile reinforced geopolymer mortar. Eng. Struct..

[39] Tamburini S., Natali M., Garbin E., Panizza M., Favaro M., Valluzzi M.R. (2017). Geopolymer matrix for fibre reinforced composites aimed at strengthening masonry structures. Constr. Build. Mater..

[40] Najm H., Secaras J., Balaguru P. (2007). Compression Tests of Circular Timber Column Confined with Carbon Fibers Using Inorganic Matrix. J. Mater. Civ. Eng..

[41] Zhang H.-Y., Hao X., Fan W. (2016). Experimental Study on High Temperature Properties of Carbon Fiber Sheets Strengthened Concrete Cylinders Using Geopolymer as Adhesive. Procedia Eng..

[42] Kurtz S., Balaguru P. (2001). Comparison of Inorganic and Organic Matrices for Strengthening of RC Beams with Carbon Sheets. J. Struct. Eng..

[43] Le Chi H., Louda P., Periyasamy A.P., Bakalova T., Kovacic V. (2018). Flexural Behavior of Carbon Textile-Reinforced Geopolymer Composite Thin Plate. Fibers.

[44] Chi H.L.E., Louda P. (2020). Flexural performance evaluation of various carbon fibre fabric reinforced geopolymer composite. Ceramics-Silikáty.

[45] Engineering M., Safi S., Zadhoush A., Ahmadi M. (2017). Flexural and Charpy impact behaviour of epoxy/glass fabric treated by nano-SiO_2_ and silane blend. Plast. Rubber Compos..

